# Supplementary material for: Patterns of Intron Gain and Loss in Fungi
Source: PLoS Biol. 2004 Nov 30;2(12):e422. doi: 10.1371/journal.pbio.0020422 (PMC532390; doi:10.1371/journal.pbio.0020422)
Supplement: Table S1 — Also available at http://genes.mit.edu/NielsenEtAl/. (4.3 MB ZIP). [file pbio.0020422.st001.zip › NielsenEtAl/html/1261.html]

AN4645.1.NCU01356.1.MG09545.1.FG10018.1


```
 CLUSTAL W (1.82) Multiple Sequence Alignments - Introns Inserted


Sequence 1: MG09545.1	440 aa
Sequence 2: FG10018.1	440 aa
Sequence 3: AN4645.1	473 aa
Sequence 4: NCU01356.1	541 aa
Alignment Length: 607 aa
Number Identitical Residues: 142 aa
Alignment Score (without introns) 7600


MG09545.1 	------------~-------------------------MA-~--IKDIPKLNRNSIRTLR
NCU01356.1	------------~-----------------------MFFTK~PRLHRIITHNLHHLLSP-
FG10018.1 	------------~-------------------------MAP~SWLNDIRRINRNNFATLR
AN4645.1  	MLPCVHGHLMSC2IRVRASTQLLTPTPAYLQRTLQGFKLRK0MTLQSIRSITEYNISTFR
          	  ..  .   :.     :::.  :.:.:  . : ..  :     :: *   .   : :  

MG09545.1 	HDPLSEISGALGDLGTLLPLMIALALQSSISLSTTLVFSGIFNVLTGIVFGIPLPVQPMK
NCU01356.1	HLLLSEISGSLGDLGTLLPLLLALSLQGSIDLPSTLLFSGLFNILTGLVFGVPLPVQPMK
FG10018.1 	AAPWAELSGSLGDLGTLLPLMIALTAQGSIDLGSTLVFTGIFNILTGAFYGIPLPVQPMK
AN4645.1  	RHYVSEIAGSLGDLGTFLPIALALAANGTVSLASTLIFSGLFNILTGLFFGIPLPVQPMK
          	    :*::*:******:**: :**: :.::.* :**:*:*:**:*** .:*:********

MG09545.1 	~AIAAAAIAEHTSLRQTVAAGGLVSAAVLILSATGLLRRATRLIPVPVAKGIQFGAGLSL
NCU01356.1	~AIAAASLQENADLETTVAAGAWVGFAVLLLGGTGGLKRVMRWVPGAVVRGVQVGAGMSL
FG10018.1 	0AIASAAIQNHSSMGVVTAAGQWVGAAVLVMSVTGLLRWVVRVVPLPVVKGIQLGAGLSL
AN4645.1  	~AIAAVAIARSFSPGSIAAAGIFVAAVLFLGSITGLLQWFTRVVPIPVVKGIQVGAGLSL
          	 ***:.:: .  .    .***  *. .::: . ** *:   * :* .*.:*:*.***:**

MG09545.1 	VISAGSSLLQPLHWLHP-------------ILDNRLWAFGALVGLVV-------------
NCU01356.1	VVAAGGGMVRPLGWLWTPEENENGHGGLGEWLDSRALAVLAFGGLVVGLGQQQQQQQQSG
FG10018.1 	ILGAGSSLLQPLHWAHP-------------ALDNRIWALVAFLVLVG-------------
AN4645.1  	VMAACTTLHG-LGWTHPS------------WADNRLWAIGVFVALLL-------------
          	::.*   :   * *  ..              *.*  *. .:  *:              

MG09545.1 	---------THRFR-RVPYALIVFILGTAFAAVAVATSG--------------RNLRLPG
NCU01356.1	EKPQERRKKRSKMPVQVPYALVLFLVGIMFAVVRVSLSKDSPQSPPPPPHDQPTNSAPPW
FG10018.1 	---------TQKLP-RFPYALHFFILALVLAFVKVTAS----------------HESLPW
AN4645.1  	---------TNSTPKRLPYALVVFIIGVVLAIIRSSLK-----------------SNLPS
          	               :.**** .*::.  :* :  : .                    * 

MG09545.1 	VAPWVPFLVLP--AWTS---PVAISMAVAQLPLTTLNSVIAVSALSADLLP---------
NCU01356.1	TWIWNPLNHIHPKVFRSLLNPQALSMAIAQLPLTTLNSIIAASALASDLFPPDSYPQLYA
FG10018.1 	FYAWRPRFSMP--RWIGNGDSPALWMAIGQLPLTTLNSIIAVTALSHDLLP---------
AN4645.1  	FSIWHPSIVIP--VGSEWS-EGAVDAGLGQLPLTTLNSVVAVVHLAADLLP---------
          	   * *   :            *:  .:.*********::*.  *: **:*         

MG09545.1 	----------------------------------NLPTPT--------VTEMGLSVASMN
NCU01356.1	DDESSDSPLSPSPSASSSSLSSAPPQTPSAETPKPLSSPTSAEEGPVPLTPLSLSISAMN
FG10018.1 	----------------------------------ELPTPS--------VTSIGISVAMMN
AN4645.1  	----------------------------------SVPTPS--------VTAIGLSVSIMN
          	                                   :.:*:        :* :.:*:: **

MG09545.1 	LIGCWFGAMPVCHGAGGLAAQHRFGARSGASIIILGIFKILLGLIL-GETLLDLLDRFPH
NCU01356.1	LLSAPFGCMPLCHGSGGLAAQHRFGARSGTSIILLGLTKFLLGLFFPGPGLLGLLGKFPK
FG10018.1 	LTSTWFGSMPVCHGAGGLAAQYRFGARSGSSIIMLGIFKLALGLFF-GETLVDLLRHYPK
AN4645.1  	LIGVWFGAMPVCHGSGGLAAQYRFGARSGASVVFLGVCKLVLGLVF-GESLVNLLHRFPK
          	* .  **.**:***:******:*******:*:::**: *: ***.: *  *:.** ::*:

MG09545.1 	ALLGIMVLASGLELAGVGQSLN-HGAADLWESSMEGNAPEALSTRIHRSLSEEERAERWS
NCU01356.1	AFLGVMVLGAGVELARVG----------------------------VRNVEGEEQDR--M
FG10018.1 	SLLGIMVVAAGLELAKVGNSLN-QGASDLWHTASG------QGPRRQRDLSEDERLERWT
AN4645.1  	ALLAVMVIAAGLELVRVGESLNTSGARDLGRQVED-------ESGEQVHLSEEERNKRWM
          	::*.:**:.:*:**. **.: .: .: .               .     :. :*: .   

MG09545.1 	VMLITTAGILAFKNDAVGFAAGMLSHWTYRAAQHWGRGTG----------------ERQP
NCU01356.1	VMLMTAGTILAFKNDGVGFLAGMGCYGGFRVAAWLGGGTEKGRGGEQGLLGEEEEEEEQG
FG10018.1 	VMLMTTAGILAFRNDAVGFFAGMLCHGAYRLSERLAERYS----------------HRIS
AN4645.1  	VMMVTVGLLVGFRNDAVGFVAGMLCHWSFELPALIHR-------------------ARHR
          	**::*.. ::.*:**.*** *** .:  :. .                         .  

MG09545.1 	LISRPFH---
NCU01356.1	RVDEESPLLR
FG10018.1 	PVEREALLR-
AN4645.1  	WSERRVRLP-
          	  ..
```
